# Genome-Wide Genetic Architecture for Common Scab (*Streptomyces scabei* L.) Resistance in Diploid Potatoes

**DOI:** 10.3390/ijms26031126

**Published:** 2025-01-28

**Authors:** Bourlaye Fofana, Braulio Jorge Soto-Cerda, Mohsin Zaidi, David Main, Sherry Fillmore

**Affiliations:** 1 Charlottetown Research and Development Centre, Agriculture and Agri-Food Canada, 440 University Avenue, Charlottetown, PE C1A 4N6, Canada; mohsin.zaidi@agr.gc.ca (M.Z.); david.main@agr.gc.ca (D.M.); 2 Departamento de Ciencias Agropecuarias y Acuícolas, Universidad Católica de Temuco, Rudecindo Ortega 02950, Temuco 4781312, Chile; bsoto@uct.cl; 3 Núcleo de Investigación en Producción Alimentaria, Facultad de Recursos Naturales, Universidad Católica de Temuco, Rudecindo Ortega 02950, Temuco 4781312, Chile; 4 Kentville Research and Development Centre, Agriculture and Agri-Food Canada, 32 Mian Street, Kentville, NS B4N 1J5, Canada; sherry.fillmore@agr.gc.ca

**Keywords:** diploid potato, common scab, *Streptomyces scabei*, disease resistance, GWAS, candidate genes

## Abstract

Most cultivated potato (*Solanum tuberosum*) varieties are highly susceptible to common scab (*Streptomyces scabei*). The disease is widespread in all major potato production areas and leads to high economic losses and food waste. Varietal resistance is seen as the most viable and sustainable long-term management strategy. However, resistant potato varieties are scarce, and their genetic architecture and resistance mechanisms are poorly understood. Moreover, diploid potato relatives to commercial potatoes remain to be fully explored. In the current study, a panel of 384 ethyl methane sulfonate (EMS)-mutagenized diploid potato clones were evaluated for common scab coverage, severity, and incidence traits under field conditions, and genome-wide association studies (GWASs) were conducted to dissect the genetic architecture of their traits. Using the GAPIT-MLM and RTM-GWAS statistical models, and Mann–Whitney non-parametric *U*-tests, we show that 58 QTNs/QTLs distributed on all 12 potato chromosomes were associated with common scab resistance, 52 of which had significant allelic effects on the three traits. In total, 38 of the 52 favorable QTNs/QTLs were found to be pleiotropic on at least two of the traits, while 14 were unique to a single trait and were found distributed over 3 chromosomes. The identified QTNs/QTLs showed low to high effects, highlighting the quantitative and multigenic inheritance of common scab resistance. The QTLs/QTNs associated with the three common scab traits were found to be co-located in genomic regions carrying 79 candidate genes playing roles in plant defense, cell wall component biosynthesis and modification, plant–pathogen interactions, and hormone signaling. A total of 61 potato clones were found to be tolerant or resistant to common scab. Taken together, the data show that the studied germplasm panel, the identified QTNs/QTLs, and the candidate genes are prime genetic resources for breeders and biologists in breeding and targeted gene editing.

## 1. Introduction

Common scab (*Streptomyces scabies*)*,* the causal agent of common scab disease in potato (*Solanum tuberosum* L.) is widespread worldwide [1,2,3,4], affecting all major potato production areas, and leads to high food waste and economic losses [5]. As per a survey conducted in 2003 across Canada, eighty two percent (82%) of the farmers surveyed had common scab problems on their farms, with an estimated economic loss of Ca$ 15 to 17 million [6]. Worldwide, despite the application of integrated agronomic and cultural practices such as soil pH and moisture control, crop rotation, seed treatment, and the use of tolerant cultivars whenever available [7,8,9,10,11], scab incidence is still problematic in most production systems [2] and as personal communication from Robert Coffin(Retired potato breeder from Cavendish Farms, PE, Canada, and current private potato breeder, Privar Farm Inc, Trenton, ON, Canada). Varietal resistance to common scab is regarded as the most sustainable and environmentally friendly option [12,13]. In this context, potato clones with introgressed common scab resistance have been reported [14], but more is needed to be achieved for highly resistant cultivars [15]. Wild relatives of potato have considerably contributed to cultivar development, and the cultivated diploid potatoes derived from *S. tuberosum* Phureja have been reported to be good sources of common scab resistance [2,16]. Historical sources for common scab resistance in potatoes included the German potato cultivar Hindenburg (HB) [17,18], which has been used in many breeding programs [2,12]. It has been suggested that scab resistance in potato is a polygenic trait, controlled by a small number of genes [2,19], which are still currently unknown, and the resistance mechanisms remain unclear [2,20], while no molecular markers linked to the traits have yet been reported [21].

The disease cycle, its pathogenicity factors and symptoms, and the cellular infection mechanisms of the scab’s phytotoxin, thaxtomin A, have been extensively investigated and reviewed [2,9,20,22,23,24,25,26,27,28,29]. Recently, the metabolic pathways potentially involved in the potato plant’s resistance to common scab diseases have started being disclosed [10,20,30,31,32,33,34,35], but are not fully understood [2,15]. Moreover, the genetic architecture of common scab resistance is not well established. Currently, only a few studies have attempted to uncover the genetic architecture of common scab resistance loci in potato, mainly using biparental QTL mapping [15,19,36,37], and GWASs on germplasm panels [3,38]. These studies have led to fragmentary conclusions, with no clear agreements between the data for the QTNs/QTLs locations [3,37,38]. Recently, a genomic selection was conducted, and heritability estimates for common scab resistance were predicted from 370 potato genotypes [39]. Commercially cultivated potatoes are tetraploid and have complex allele dosage effects that affect gene expression [40,41], making their genetics more complex compared to diploid potatoes. To date, diploid potato relatives remain untapped in potato germplasm for their full potential as abiotic and biotic stress resistance sources [42,43]. To our knowledge, using diploid potato germplasm panel, very few [36] GWASs have been reported to map the genetic architecture of common scab resistance and its associated genes. It is known that cultivated diploid potatoes and their wild relatives to cultivated tetraploid potatoes have co-evolved in their natural habitats while coping with biotic and abiotic stress [44,45,46], and many sources of resistance have been identified in both cultivated landraces and wild species [43,47,48]. In our previous studies, we developed an ethylmethane sulfonate (EMS)-mutagenized diploid potato germplasm collection derived from seven crosses involving one female and seven pollen donors in bi-parental crosses, and reported a high genetic diversity and novel phenotypic variants [49,50]. Further, using genome-wide association studies, we uncovered the genetic architecture for plant maturity and drought tolerance in a 384 EMS-mutagenized diploid potato germplasm panel grown under field conditions [51]. But the genetic architecture for common scab resistance in this panel is still unknown. We hypothesize that the EMS-mutagenized diploid panel will show a genomic architecture associated with common scab traits.

The objectives of this study were (1) to assess the genetic architecture of three common scab phenotypic traits in the 384 EMS-mutagenized diploid potato germplasm panel grown under scab-infested field conditions, and (2) to identify candidate genes associated with scab-resistance traits. Using two GWAS statistical models and a non-parametric test, QTNs/QTLs associated with the common scab traits were identified, and we showed that 38 QTNs/QTLs located on eight chromosomes were pleiotropic and affected at least two common scab traits. Three chromosomes were unique in carrying QTNs/QTLs affecting a single trait. A total of 34, 30, and 15 candidate genes were found to be co-located with the QTNs/QTLs associated with common scab coverage, severity, and incidence traits, respectively. The data show that common scab inheritance in the studied diploid potato germplasm panel is quantitative and multigenic, and the identified QTLs/QTNs and genes are prime genetic resources for breeders and biologists in conventional breeding and targeted gene editing.

## 2. Results

### 2.1. Trait Phenotypic Distribution

**Figure 1:**
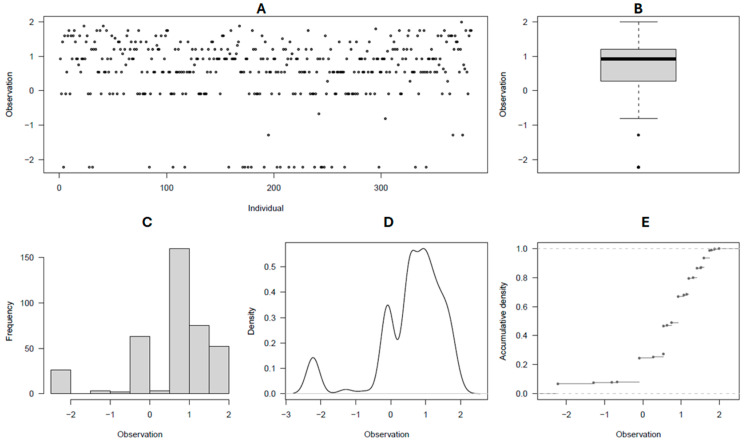
(**A**–**E**) Common scab severity distribution among the 384 germplasm panel; (**A**) spatial visual distribution of individuals for scab disease severity rating classes; (**B**) scatter box plot of average distribution; (**C**) frequency distribution of individuals for scab severity ratings; (**D**) density plot of individuals for scab severity ratings; and (**E**) cummulative density distribution for scab severity ratings.

**Figure 2:**
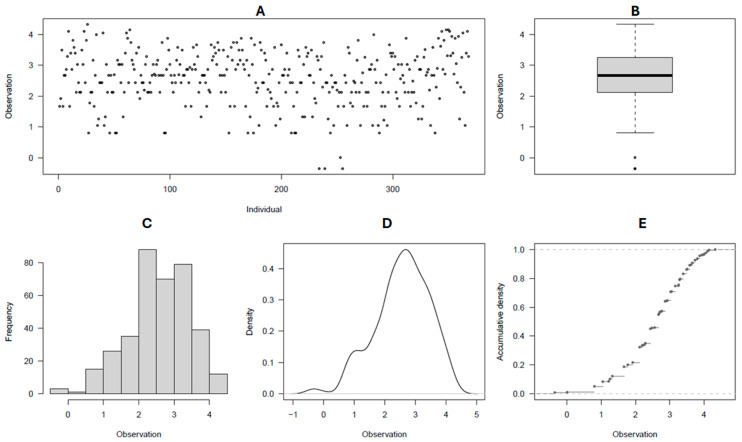
(**A**–**E**) Common scab coverage phenotype distribution among the 384 germplasm panel; (**A**) spatial visual distribution of individuals for scab disease coverage rating classes; (**B**) scatter box plot of average distribution; (**C**) frequency distribution of individuals for scab coverage ratings; (**D**) density plot of individuals for scab coverage ratings; and (**E**) cummulative density dstribution for scab coverage ratings.

**Figure 3:**
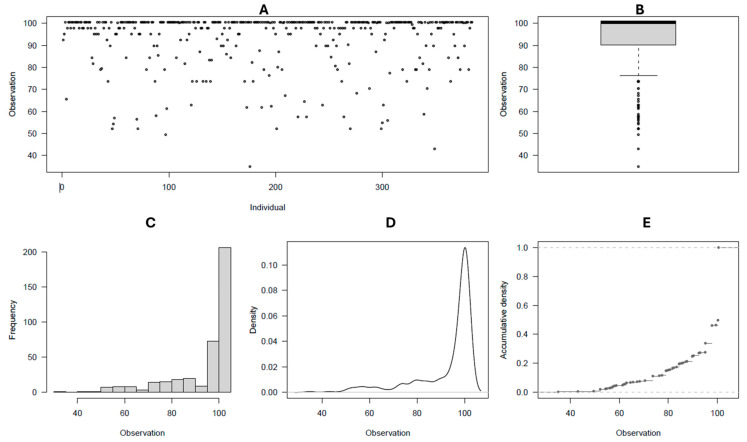
(**A**–**E**) Common scab incidence distribution among the 384 germplasm panel; (**A**) spatial visual distribution of individuals for scab disease incidence rating classes; (**B**) scatter box plot of average distribution; (**C**) frequency distribution of individuals for scab incidence ratings; (**D**) density plot of individuals for scab incidence ratings; and (**E**) cummulative density disstribution for scab incidence ratings.

By rating the common scab reactions as two datasets (2021 and 2022, and 2021 to 2023), the disease severity and surface coverage of affected tubers followed a fairly normal phenotypic distribution (Figure 1A–E and Figure 2A–E), whereas the disease incidence was found to be skewed towards the high incidence of scab in the population (Figure 3A–E, Table 1). The observed grand means were 98.8 ± 1.6, 2.46 ± 0.58, and 16.51 ± 8.61, for incidence, severity, and surface coverage in the 2021–2022 dataset, respectively, whereas the same estimators were 86.65 ± 11.94, 1.97 ± 0.62, and 11.58 ± 5.86 in the 2021–2023 dataset. Overall, for each scab rated trait and in each dataset, significant differences (*p* < 0.001) were observed between the potato clones for their reaction to common scab, and a consistent range of variations were observed in the two datasets for the three traits assessed. Most of the potato clones evaluated (97–99%, 55–76%, and 47–65%) were scored as medium to high incidence, severity, and scab coverage, respectively, in the two datasets (Table 1). Overall, the 61 (16%) potato clones evaluated, including the resistant check Hindenberg, showed 0–5% scab severity (Supplementary Figure S1).

**Figure 4:**
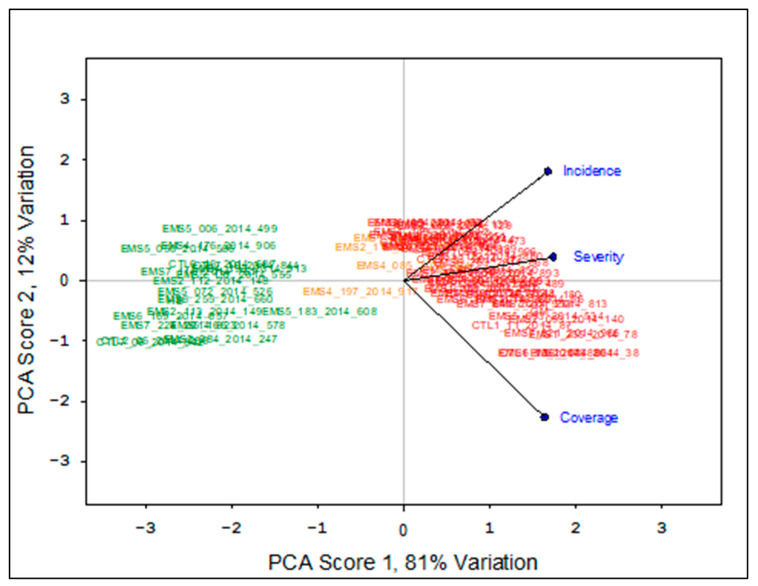
Principal component analysis of the 2021–2023 dataset depicting the differentiation among the 68 clones most contrasting for common scab reaction. Three groups can be observed. Green: low rating for incidence, severity, and surface coverage. Red: high rating for incidence, severity, and surface coverage. Orange: low to medium rating. The red grouping is closely associated with high incidence, severity, and coverage. Note that the sample IDs may be found overlaped and are not intended to be readable in each group.

Using the means from the mixed model analysis, hierarchical clustering was performed to generate the most differentiated clustered groups reflecting the common scab reactions. On each dataset a PCA using the correlations of Euclidian distances was performed on the differentiated cluster groups. A total of 79 and 68 potato clones were found to be clearly discriminated for scab reaction in the 2021–2022 and 2021–2023 datasets with 60% and 81% variation, respectively (Figure 4).

### 2.2. Identification of QTNs/QTLs Associated with Common Scab Traits

**Figure 5:**
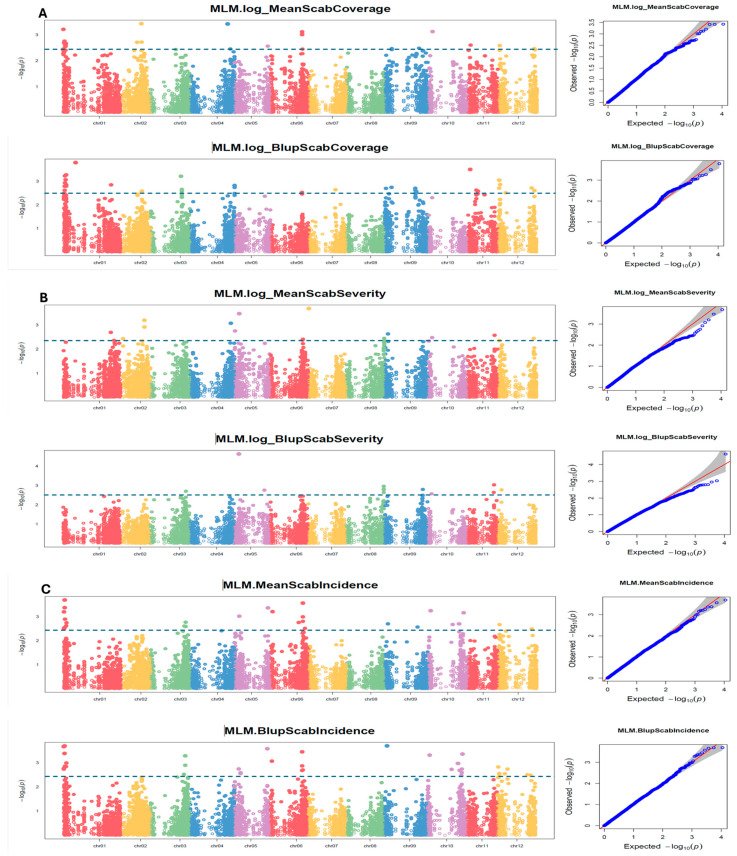
(**A**–**C**) Manhattan and Q-Q plots showing QTNs/QTLs and chromosomal regions associated with common scab traits using the GAPIT-MLM. Each panel corresponds two datasets (mean and BLUP). (**A**) Coverage mean and BLUP; (**B**) severity mean and BLUP; and (**C**) incidence mean and BLUP. The dotted line indicates the cut off −log_10_(*p*) < 2.5. The six boxed inserts on the right present the quantile–quantile (Q-Q) plot for each Manhattan plot, showing a well-fitted GWAS model, with minimal artifact bias from −log_10_(*p*) values > 2.5. The blue dots represent the *p*-values observed from the genomic association study. The red line is the expected distribution of *p*-values when there is no association under the null hypothesis. It acts as a reference line to assess if the data deviate significantly from the expected distribution. Since most tested SNPs may not be associated with the trait, the majority of blue dots in the Q-Q plot should fall on the red line, indicative of a good fit to the null hypothesis, as shown. The deviations from the red line suggest potential significant associations. The gray area indicates the 95% confidence interval under the null.

The SNP discovery and genetic structure of the population have been previously reported by Fofana et al. [51]. Using the same SNP dataset and population structure as previously reported, a 11,605 SNP set was tested for association with each of three independent 2021–2022 phenotypic datasets for surface coverage, scab severity, and scab incidence traits. Using the GAPIT MLM GWAS, a total of 83, 70, and 53 significant (*p* < 0.05) SNPs were found to be associated with scab coverage, severity, and incidence, respectively (Supplementary Table S1a–c). Significant (*p* < 0.05) SNPs were observed on all 12 chromosomes, for each of the three scab traits, except for scab incidence, for which GAPIT did not detect significant SNPs on chromosome 11 (Supplementary Table S1c). Chromosomes 1 and 6 showed the most SNPs associated with the three traits. The quantile–quantile (Q-Q) plot showed a well-fitted GWAS model, with minimal artifact bias from −log_10_(*p*) values > 2.5 (Figure 5A–C).

#### 2.2.1. Scab Coverage

The 83 significant SNPs (FDR < 0.05) found to be associated with scab coverage were distributed over all 12 chromosomes (Supplementary Table S1a, Figure 5A). Chromosomes 1, 2, 4, 6, and 10 showed more SNPs with −log_10_(*p*) values > 2.5. The quantile–quantile (Q-Q) plot indicates a fairly well-fit GWAS model, and minimal artifact bias from −log_10_(*p*) values > 2.5 (Figure 5A). The mapped significant SNPs showed large effects on scab coverage, varying from -0.30 to 0.32 effect on coverage (Supplementary Table S1a).

Similarly, by performing haploblock-based RTM-GWAS analysis using the same SNPs and phenotypic datasets, a total of 58 QTNs/QTLs were detected on the 12 chromosomes for the three scab traits. Of these 58 QTNs/QTLs, 13 QTNs, and 12 haplo-block QTL loci were found to be associated with the scab coverage trait, of which 3 (chr01_3114493-3114575, chr04_4988793, Block_chr06_48442356) were found in at least two datasets (Supplementary Table S1d). By applying the second FDR < 0.05 threshold, only two significant QTNs were detected on chromosomes 1 and 4 as highly significant (Supplementary Figure S2A,B). Taken together, 13 QTN markers and 10 of 12 haploblock loci were detected using the two complementary statistical models to be strongly associated with the scab coverage trait on 10 of the 12 potato chromosomes (Supplementary Table S1d).

#### 2.2.2. Scab Severity

The GAPIT model detected 70 significant SNPs (FDR < 0.05) associated with scab severity that were distributed on all 12 chromosomes (Supplementary Table S1b, Figure 5B). Chromosomes 2, 4, 5, and 7 showed more SNPs with −log_10_(*p*) values > 2.5. The quantile–quantile (Q-Q) plot indicates a fairly well-fit GWAS model, and minimal artifact bias from −log_10_(*p*) values > 2.5 (Figure 5B). The mapped significant SNPs showed large effects on scab coverage, varying from −5.40 to 4.01 effect on severity (Supplementary Table S1b).

RTM-GWAS haploblock analysis was also performed and allowed us to identify 9 QTNs and 12 haplo-block QTL loci associated with scab severity (Supplementary Table S1d). As for the GAPIT-MLM GWAS, the 21 QTNs/QTLs detected by the RTM-GWAS model were distributed on 10 of the 12 potato chromosomes. Of the 21 QTNs/QTLs, 4 (Block_chr06_1662317-1662359, chr06_48682020, chr08_58892718_58892767, chr12_1934225_1934235) were found in at least two datasets, and 20 of the 21 QTNs/QTLs were detected using the two GWAS models (Supplementary Table S1d). By applying the second FDR < 0.05 threshold, five significant QTNs were detected on chromosomes 1, 3, 8, and 11 to be highly significant (Supplementary Figure S3A,B). Taken together, nine QTN markers and eleven haploblock loci were detected using two complementary statistical models to be strongly associated with the scab severity trait on nine of the potato chromosomes.

#### 2.2.3. Scab Incidence

A total of 53 significant SNPs (FDR < 0.05) were found to be associated with the scab incidence trait by the GAPIT model, and were distributed on 11 of the 12 potato chromosomes (Supplementary Table S1c, Figure 5C). Chromosomes 1, 5, 6, and 10 showed more SNPs with −log_10_(*p*) values > 2.5. The quantile–quantile (Q-Q) plot indicates a fairly well-fit GWAS model, and minimal artifact bias from −log_10_(*p*) values > 2.5 (Figure 5C). The mapped significant SNPs showed large effects on scab coverage, varying from a −5.57 to 5.07 effect on incidence (Supplementary Table S1c).

By performing the RTM-GWAS haploblock analysis, a total of two QTNs and nine haplo-block QTL loci were associated with scab incidence (Supplementary Table S1d). The 11 QTNs/QTLs herein detected were found distributed on six of the twelve potato chromosomes. Of the 11 QTNs/QTLs, 2 haploblocks (chr06_29687284_29687343, chr-10_50975501_50975603) were found in at least two datasets, and 4 of the 11 QTNs/QTLs were detected via the two GWAS models (Supplementary Table S1d). By applying the second FDR < 0.05 threshold, 12 significant QTNs were detected on chromosomes 1, 2, 4, 5, 6, 7, and 10 (Supplementary Figure S4A,B).

In total, of the 58 QTNs/QTLs detected by the GAPIT MLM and RTM-GWAS models, 10 QTNs/QTLs associated with the scab traits were found in at least two datasets, and sixteen QTNs/QTLs were found to be pleiotropic on at least two of the scab traits. Of the sixteen, six QTNs/QTLs located on chromosomes 3, 4, 5, 9, and 11 were pleiotropic on all three scab traits (Supplementary Table S1d, Table 2).

Using Mann–Whitney non-parametric *U*-tests on the 58 significant QTNs/QTLs detected by the GAPIT MLM and RTM-GWAS models, we found associations with the three scab traits, where 52 had significant allelic effects across 11 of the 12 chromosomes. A total of nine QTNs and 11 haploblocks showed significant allelic effects in reducing scab coverage across eight chromosomes, six QTNs and 10 haploblocks had allelic effects on scab severity across nine chromosomes, and five QTNs and 11 haploblocks had allelic effect on reducing scab incidence across nine chromosomes. Only chromosome 2 did not show QTNs/QTLs with significant allelic effects. The allelic effects contributing to reducing scab traits varied from −0.71 to −0.18, −0.64 to −0.15, and −7.7 to −1.9 for coverage, severity, and incidence, respectively (Table 2).

### 2.3. Favorable Alleles Affecting the Traits

**Figure 6:**
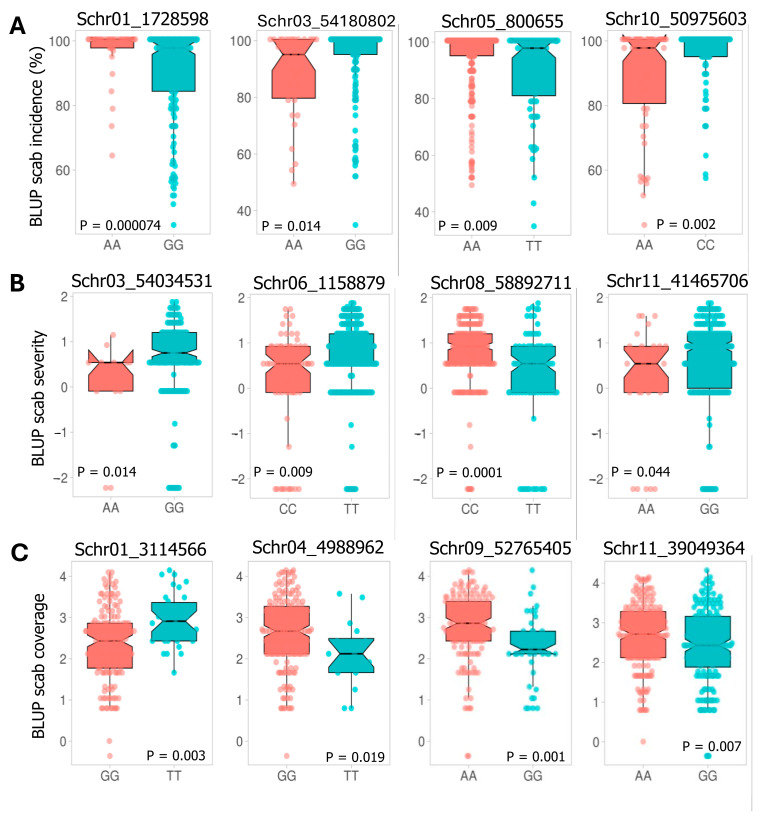
(**A**–**C**) Violin plots illustrating the phenotypic differences between potato genotypes carrying different alleles of the significant SNPs. (**A**) Scab incidence, (**B**) scab severity, and (**C**) scab coverage. Means and standard variations for each SNP allele are shown. Statistical differences between alleles were tested using the Mann–Whitney non-parametric U test (*p* < 0.05).

The phenotypic differences between resistant and susceptible potato clones were obvious for coverage, severity, and incidence (Supplementary Figure S1). For all the scab traits, Mann–Whitney non-parametric *U*-tests showed that 52 QTNs/QTLs had allelic effects and allelic variants that contribute to reducing scab coverage, severity, and incidence, with 20, 16, and 16 QTNs/QTLs, respectively (Table 2). A subsample allelic effect from 12 QTNs/QTLs, among the 52 QTNs/QTLs identified and shown to affecting the traits, is shown in Figure 6A–C.

**Figure 7:**
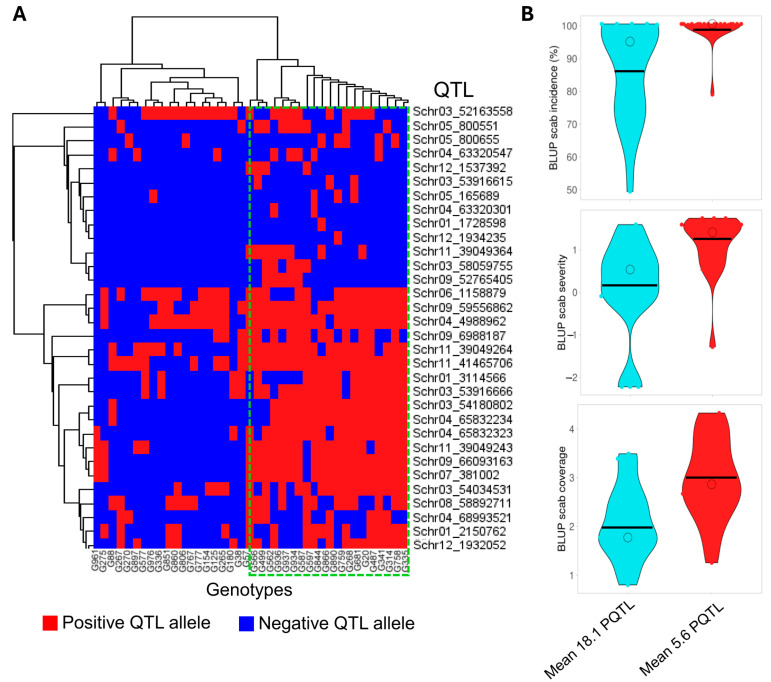
(**A**,**B**) Heatmap and violin plots displaying positive and negative allelic effects among 40 potato clones with differential common scab reactions. (**A**) Heatmap displaying the distribution of 32 unique positive QTL (PQTL) alleles among 20 high and 20 low potato clones carrying more or fewer PQTLs; (**B**) violin plots illustrating the mean of favorable PQTL for the best potato genotypes carrying 18 PQTLs and the worst genotypes carrying 5.6 PQTLs for each common scab trait.

By performing a heatmap distribution analysis of positive QTL (PQTL) alleles among 20 high and 20 low 5% genotypes, 32 unique PQTLs clearly discriminated between the 40 potato clones based on the number of PQTLs they carry (Figure 7A). For each scab trait, the best genotypes were found to harbor an average of 18 PQTL with a range from 17 to 19, while the most susceptible subset had an average of 5.6 with a range from 0 to 8, highlighting the significant negative correlations (*p* < 0.01) of −0.48, −0.51, and −0.57 between the number of favorable PQTLs and scab incidence, scab severity, and scab coverage, respectively. This indicates that as the number of PQTLs increases, the scab incidence, severity, and coverage decrease (Figure 7B).

### 2.4. Candidate Genes Associated with Common Scab Traits

By scanning the 20 kb genomic regions carrying the 58 QTNs/QTLs associated with three scab traits, 79 candidate genes were found to be associated with the scab traits, of which 34, 30, and 15 candidate genes were associated with the scab coverage, severity, and incidence traits, respectively (Supplementary Table S2). Genes were found to be associated with the scab coverage trait, included in plant defense (Soltu.DM.01G004470.1; Soltu.DM.06G000830.1; Soltu.DM.07G000050.1; Soltu.DM.09G018920.1; Soltu.DM.08G026440.1; Soltu.DM.10G021860.1; Soltu.DM.10G021930.1; Soltu.DM.12G002390.1), hormonal signaling (Soltu.DM.03G027480.1; Soltu.DM.04G025270.1; Soltu.DM.03G034600.1; Soltu.DM.10G021860.1; Soltu.DM.11G021470.1), cell wall biosynthesis and modification (Soltu.DM.04G004640.1; Soltu.DM.09G018910.1; Soltu.DM.01G001840.1; Soltu.DM.01G001850.1; Soltu.DM.03G029620.1; Soltu.DM.06G025660.1), cell membrane transport (Soltu.DM.07G000080.1), cell differentiation and proliferation (Soltu.DM.03G034660.1; Soltu.DM.11G021480.1), and TFs (Soltu.DM.03G027480.1; Soltu.DM.12G002380.1). Interestingly, the genomic regions carrying the QTNs/QTLs, including Schr01_1728598, Schr03_52163558, Block_chr03_58019530_58059755, Schr04_4988962, Schr09_6988187, and Schr09_52765405, shown to have significant allele effects on scab coverage, harbored candidate genes involved in stress response (Soltu.DM.01G001680.1), transcription factor activity (Soltu.DM.03G027480.1), hormone signaling (Soltu.DM.03G034600.1, Soltu.DM.09G007360.1), cell wall degradation, and expansion (Soltu.DM.04G004640.1, Soltu.DM.09G018910.1), and in plant–pathogen interaction and immune responses (Soltu.DM.09G018920.1) (Supplementary Table S2).

The 30 candidate genes co-located with QTNs/QTLs associated with scab severity include genes involved in disease resistance (Soltu.DM.06G001240.1; Soltu.DM.06G001240.1; Soltu.DM.12G002390.1; Soltu.DM.12G002410.1), plant–pathogen interaction (Soltu.DM.09G018920.1), signal transduction (Soltu.DM.05G015520.1; Soltu.DM.03G029780.1; Soltu.DM.12G026650.1), stress response (Soltu.DM.08G029880.1; Soltu.DM.01G002950.1), cell wall modification (Soltu.DM.04G004640.1; Soltu.DM.07G000050.1; Soltu.DM.05G000090.1; Soltu.DM.05G001020.1; Soltu.DM.09G023660.1; Soltu.DM.09G023670.1), hormone signaling (Soltu.DM.12G026660.1), cell proliferation and elongation (Soltu.DM.06G008090.1; Soltu.DM.11G021480.1; Soltu.DM.12G002380.1), and transcription factor activities (Soltu.DM.01G002070.1; Soltu.DM.12G002380.1; Soltu.DM.12G026660.1). The QTNs/QTLs Block_chr03_54180732_54210225, Block_chr05_800551_800594, chr07_381002, and Block_chr08_58892718_58892767, which had a significant allele effect on scab severity, were associated with candidate genes involved in calcium signaling (Soltu.DM.03G029780.1), cell wall biosynthesis (Soltu.DM.05G001020.1), cell membrane pumping and transport (Soltu.DM.07G000080.1), endonuclease activity and RNA silencing (Soltu.DM.07G000050.1), stress-activated malate signaling (Soltu.DM.08G029880.1), and disease resistance (Soltu.DM.12G002390.1) (Supplementary Table S2).

Scab incidence was associated with 15 genes, including disease resistance (Soltu.DM.04G034340.1; Soltu.DM.12G002390.1; Soltu.DM.12G002410.1), signal transduction (Soltu.DM.10g019550.1), wound stress response (Soltu.DM.09G029980.1), cell wall modification (Soltu.DM.03G029620.1; Soltu.DM.04G031750.1; Soltu.DM.09G030000.1), cell membrane hormonal signaling and cell proliferation, and elongation (Soltu.DM.11G000400.1; Soltu.DM.10G019550.1; Soltu.DM.10G019560.1). As for scab coverage and scab severity, among the 16 QTNs/QTLs detected with a significant allelic effect on scab incidence, 3 QTNs/QTLs (chr04_65832246, Block_chr10_50975501_50975603, Block_chr11_39049243_39049264) were associated with genes playing roles in disease resistance (Soltu.DM.04G034340.1), signaling and cell wall expansion (Soltu.DM.10G019560.1, Soltu.DM.10G019550.1), and cell membrane lipid metabolism (Soltu.DM.11G019630.1). Of interest were the overlapping genes found to be associated with at least two of the traits, including seven genes between coverage and severity (Soltu.DM.01G002950.1; Soltu.DM.04G004640.1; Soltu.DM.07G000050.1; Soltu.DM.07G000080.1; Soltu.DM.11G021480.1; Soltu.DM.12G001870.1; Soltu.DM.05G015540.1), two between coverage and incidence (Soltu.DM.03G029620.1; Soltu.DM.11G019630.1), three between severity and incidence (Soltu.DM.04G032530.1; Soltu.DM.04G032550.1; Soltu.DM.12G002410.1), and two between the three traits (Soltu.DM.12G002380.1; Soltu.DM.12G002390.1). The common features of these genes are their roles in disease resistance, cell wall and membrane metabolism and fortification, and cell development, and as transcription factors (Supplementary Table S2).

### 2.5. Comparison Between GWAS-Associated and RNA-Seq Transcriptomic Gene Sets

By studying the comparative transcriptome expression of the common scab susceptible potato cultivar “Green Mountain” and the resistant cultivar “Hindenburg”, we had previously shown a contrasting transcriptome expression profile between the two cultivars, with 273 differentially expressed genes in 34 pathways (Supplementary Table S3 [34]. Here, by cross-referencing these RNAseq gene expression data with the current GWAS-detected gene set, a consistent cross-reference gene detection was observed using the two platforms (Table 3). Genes involved in plant defense and immunity, disease resistance, hormone signaling, cell wall modification and strengthening, transcription factor activities, terpenoid secondary metabolites biosynthesis, and signal transduction were found in the current GWASs and the previous RNASeq study (Table 3, Supplementary Table S3 [34]).

## 3. Discussion

Common scab disease negatively impacts potato quality and marketability [39], and causes high levels of food waste [58]. Varietal resistance to common scab and biocontrol agents that are antagonists to common scab are seen as the most sustainable and environmentally friendly strategies [5,59,60]. Currently, not many potato varieties are scab-resistant because scab varietal resistance is dependent on genotype, time, and environmental conditions [61,62]. More scab-resistant potatoes are therefore required [3,63,64,65]. In the current study, a mutagenized diploid potato germplasm panel [49,51] was evaluated under field conditions for common scab reactions, and GWASs were conducted. Here, we report a wide range of plant reactions to common scab, with 52 significant QTNs having favorable allelic effects on all the three scab traits, as well as candidate genes with known roles in disease resistance and scab pathogenesis. To our knowledge, this study is the first to report the quantitative and multigenic nature of common scab resistance in diploid potatoes.

Large genetic variations were observed in the population, with fairly normal distributions for all common scab traits herein studied. Scab symptoms have indeed previously been reported for their quantitative distribution with GxE interactions [36,66]. Field phenotyping is usually associated with GxE interactions [67,68] and in the current study, consistent scab reactions were observed over years, albeit with slight variations between years and datasets, as expected. Replicated plots for each clone per year would have been ideal. However, multi-year trials of the same clones, as performed in the current study, contributed to assessing the performance of the clones. In fact, using the same germplasm panel in the same artificilly scab-infested field over 3 years, it was expected that any genotypes with low scab incidence, severity, and surface coverage would be deemed carryiers of genetic armour againt common scab, as was the case for 61 potato clones uncovered in the current study. These clones will require further study for other agronomic traits. Moreover, since some of these clones are fertile and produce berries, they can be used as gene donors in breeding programs or be developed as diploid potato varieties with scab resistance.

It has been suggested that common scab resistance is quantitatively controlled by several genes in tetraploid potatoes [21,69], but controlled by one or a few genes in diploid potatoes [14,19,37,69]. In the current study, 58 QTNs/QTLs located on all 12 chromosomes were found to be associated with common scab resistance, as detected by two GWAS models. Indeed, as previously reported [51], two advanced GWAS statistical models, GAPIT-MLM and RTM-GWAS, were used in the current study to dissect the genetic architecture of scab resistance traits, acknowledging that each model has its strength and weakness, thus being complementary. It is established that statistical models used in GWAS analyses can have a major impact on the output data [70,71,72,73]. Single-locus GWAS statistical models including GLM and MLM, GAPIT-MLM, and GAPIT-cMLM can detect large effects QTNs, whereas multi-locus models such as mrMLM and RTM-GWAS increase the detection power of QTNs [74,75,76,77,78,79,80]. The MLMs best fit the kinship as a random effect to control the genetic background caused by the genetic relatedness among individuals in the population, theoretically correct the inflation from small polygenic effects, and efficiently control the population stratification bias [72], reduce false positives [81], and generate QTLs. Therefore, MLMs are good for QTN/QTL detection. Nonetheless, the RTM-GWAS model exhibits the lowest false discovery rate due to its very stringent correction criteria [82]. In the current study, 83, 70 and 53 QTNs were identified on all 12 chromosomes using the GAPIT-MLM for scab coverage, severity, and incidence traits, respectively, whereas 25, 21, and 12 QTNs/QTLs were detected by the RTM-GWAS model on 11, 10, and 6 chromosomes for the same three traits, respectively. The detection of fewer QTNs/QTLs by multi-locus GWAS models compared to single-locus GWAS models is common, and has been previously reported when comparing GAPIT-cMLM and mrMLMs [83], and in previous GWASs in potato using the same panel [51]. By imposing the second-stage threshold of the RTM-GWAS model, a very low number of QTNs/QTLs were detected for each of the scab coverage and severity traits (Supplementary Figures S2–S4), confirming and highlighting the stringency of the RTM-GWAS model. Previous studies have reported sparse numbers for chromosomes with genomic regions were associated with common scab resistance in potato. Altogether, using biparental QTL mapping studies [2,19,36,37] and GWASs of a germplasm panel [3,38], chromosomes 1, 2, 3, 4, 5, 7, 9, 10, 11, and 12 have been shown by six independent studies to carry QTLs associated with common scab resistance. However, each of these studies led to fragmented data, with incongruent conclusions on the genomic locations and chromosomes carrying the QTLs. In particular, only studies by Zorrilla et al. [37] and Yuan et al. [38] reported more than two chromosomes carrying QTLs associated with scab resistance, each reporting three (chromosomes 2, 4, and 12), and seven (chromosomes 1, 2, 3, 5, 6, 10, and 11), respectively. Here, using two GWAS models, we report that 6 to 11 of the 12 potato chromosomes were found to carry significant QTNs/QTLs associated with the three scab traits studied. A total of 6, 10, and 11 chromosomes were found to be associated with scab incidence, severity, and coverage, respectively, but all together, all 12 chromosomes were found to be involved (Supplementary Table S1d). Mann–Whitney non-parametric *U*-tests further confirmed that 52 of the 58 QTNs/QTLs detected by the two GWAS models had allelic and favorable effects on reducing common scab attacks. These data suggest that common scab resistance loci are widely distributed across the potato genome. Our findings are in agreement with the previous aggregate findings from the previous six independent studies where, altogether, 10 of 12 of the potato chromosomes carried QTLs associated with common scab resistance. Our current data are significant as they highlight the polygenic nature and quantitative control of common scab resistance in both diploid and tetraploid potatoes, contrasting with a previous report by Murphy et al. [14]. If it is accepted that tetraploid potatoes arose from the genome duplication of a diploid species (autopolyploidization), or as a hybrid between two diploid species (allopolyploidization) [84,85,86], the current data make more sense in that tetraploid potato might have inherited the common scab disease resistance loci from diploids [87,88,89], some of which might have been lost through selection pressure. The later assumption may possibly explain the fragmented data reported in previous QTLs and GWASs in tetraploid potatoes. Nonetheless, the current germplasm panel is derived from mutagenesis with new loci generated, making it a highly valuable population for trait studies.

Using the GWAS models, pleiotropic QTNs/QTLs were identified. These findings were further tested by the non-parametric *U*-test, and 38 QTNs/QTLs were pleiotropic on at least two of the common scab traits, suggesting their high impact on the common scab traits. The data reported here are worth significant attention because they derive from a unique and unprecedented mutagenized diploid germplasm panel. All previous mapping studies on common scab have focused on tetraploid biparental populations [2,19,36,37] and tetraploid germplasm panels [3,38]. Only Braun et al. [15] identified common scab resistance QTL on chromosome 11 in diploid potato. To our knowledge, no previous investigations have reported such a wide spectrum of QTN/QTL identification in diploid potato germplasm panel.

Potato resistance mechanisms to common scab are still unclear and not fully understood, although differential expression of candidate genes have been reported [31,34,35]. In the current study, using GWASs, a total of 34, 30, and 15 candidate genes were found to be associated with scab coverage, severity, and incidence, respectively, and were found across the genomic regions of the 12 potato chromosomes. The QTNs/QTLs located in the genes or in the vicinity of the genes associated with common scab resistance traits include genes involved in plant defense, hormonal signaling, cell wall biosynthesis and modification, cell membrane transport, cell differentiation and proliferation, transcription factor activities, disease resistance, signal transduction, cell proliferation, and elongation, some of which were found overlapping between the three common scab traits. Some of the candidate genes herein identified were previously reported through RNAseq obtained by studying a common scab-resistant cultivar (Hindenburg) and a susceptible cultivar (Green Mountain). Finding the same genes in unrelated potato clones is an indication that the detected genes are critical in the potato–common scab pathosystem, and could be prime targets for genetic improvements, notably through gene editing. It is worth noting that a maximum of 19 QTNs/QTLs were observed among the clones; hence, designing specific crosses between individuals harboring complementary QTNs/QTLs could allow for increasing scab resistance through QTL pyramiding.

Recently, da Silva Pereira et al. [36] reported a single QTL for lesion traits spanning 96 to 103 cM on chromosome 3 in tetraploid potato. The QTL interval implicated 23 genes, of which 3, including MYB transcription factor, calcium-dependent protein kinase 1 (CDPK1), and ubiquitin-protein ligase, were reported to be implicated in plant responses to biotic stress. Two of these genes (CDPK1 and ubiquitin-protein kinase) were also detected on chromosome 3 in the current GWAS study. For example, the Arabidopsis CPK1 gene mediates pathogen resistance by regulating the accumulation of salicylic acid and the expression of salicylic acid-regulated defense and resistance genes [90].

Our gene detection data expand those reported by da Silva Pereira et al. [36], with more chromosomes impacting scab resistance. The mechanisms by which common scab causes cell hypertrophy, collapse, and death during the infection process under the action of the bacterium’s thaxtomin A are fairly well known [1,11,26,27,28]. In reaction to wounding by the bacterium, the plant reacts by rapid tissue division and cell wall suberization, forming corky cells to seal the wound [32,59]. Here, genes involved in HR (LRR = Soltu.DM.12G026650.1), lignin biosynthesis and metabolism (Soltu.DM.01G001840.1, Soltu.DM.01G001850.1), cell proliferation (Soltu.DM.11G021480.1, Soltu.DM.06G001210.1), and terpenoid biosynthesis were detected in the GWASs and RNAseq. Terpenoids are known for playing roles in wound healing [91,92,93], and genes involved in their biosynthesis, along with those involved in cell fortification, were also uncovered here. These findings are well supported by the corky piths observed following scab attacks. Other genes identified here, such as calmodulin and subtilisin-like proteins, are consistently found as genes involved in this pathosystem [31,34]. Calmodulin plays key roles in the calcium channel and signaling, and calcium flux has been found to increase under the action of Thaxtomin A [2]. The role of subtilisin-like protein in plant–pathogen recognition and immune priming is known in plants [34,94]. This gene was identified in the current GWAS. Notably, disease resistance genes such as *ADR1* (Soltu.DM.04G034340.1), and genes involved in cell wall biosynthesis and modifications and hormone signaling, were also identified as having been previously reported in plant–pathogen and common scab–potato interactions [31,34,95]. All these genes concur in synergy to resistance to common scab. As previously reported [3,64,69,96], most have small phenotypic effects, but together they contribute to higher resistance levels, as shown by the heatmap analyses using the number of favorable QTL alleles in potato genotypes. Our findings strongly suggest that relevant SNP markers associated with common scab-resistance genes can be developed from the current study and that, based on the nature of polygenic inheritance, with a small effect, breeding for such a trait in tetraploid potato may be tedious, as previous reported [97]. Such difficulty may be overcome either by crossing diploid potato with tetraploid potato, or crossing two diploid potatoes, followed by diploidization, if the commercial production has to rely solely on tetraploid potatoes. Nonetheless, developing diploid potato cultivars could be promising.

In conclusion, this study dissected the genetic architecture of a diploid potato germplasm panel for QTNs/QTLs associated with common scab traits. It provided primary evidence that common scab resistance in diploid potato is quantitative and multigenic with small gene effects across all 12 chromosomes, and that multiple loci overlap (pleiotropic) between traits to contribute to resistance. The identified genetic loci can be worth studying for marker development and further detailed functional studies. The data and germplasm herein reported are prime genetic resources for breeders and biologists in conventional breeding and targeted gene editing.

## 4. Materials and Methods

### 4.1. Plant Materials and Scab Evaluation Field

The plant materials used in this study were derived from an ethyl methane sulfonate (EMS)-mutagenized diploid potato germplasm previously developed and described by our group [49]. It consisted of a 384 diploid potato germplasm panel including 47 wild types (CTL) and 337 EMS-mutagenized (EMS) clones (Supplementary Table S4), where the proportion of clones per crosses varied from 10% to 19% (Table 4), as stated by Fofana et al. [51]. The entire original collection of 814 clones [49] has continuously been propagated in the field each year since 2014 in a 5–6-hill plot without replication. All field trials were conducted under conventional agronomic practices at the Agriculture and Agri-Food Canada (AAFC) Harrington Farm (Harrington, PE, Canada). In 2021, the core 384 germplasm panel was formally established and tested in a separate field for common scab (*S. scabei*) resistance in 5–6-hill small plots without replication. The selection of the clones in the panel was previously described in Fofana et al. [51]. As checks, the German late-maturing tetraploid potato cultivar Hindenburg (HB) derived from a cross between Ismene × Jubel and expressing a high resistance to common scab [17] was used as a scab-resistant check, while the heritage late-maturing potato cultivar Green Mountain (GM) derived from the cross Dunmore × Excelsior by Alexander OH in 1885 and Shepody was used as a susceptible check.

The common scab evaluation field at the Agriculture and Agri-Food Canada Harrington Farm (PE, Canada) is a 0.712 ha field devoted to AAFC’s breeding lines under evaluation for common scab. The field was established in the 1990s (as per a personal communication from Dhuey Pratt, retired Agriculture and Agri-Food Canada farm manager). To do so, this field was chosen in the first place for its low pH. Composted manure was applied to the field in the spring of the first year and formally identified common scab-infected potatoes were spread in the fall and disk harrowed. In subsequent years, composted manure was replaced by conventional fertilizer as used in commercial farms, and scabby potato tubers were dumped over the field for at least three years to build up the initial inoculum. After three years, susceptible potatoes were planted in the field to assess a uniform distribution of scab bacterium across the field, which was achieved in 1994. Since then, this field, herein referred to as the scab field, has been used for evaluating potato breeding clones to scab, and each year, scabby potatoes are dumped and dispersed across the field to maintain high inoculum levels.

### 4.2. Common Scab Testing and Phenotyping

For this study, common scab testing was performed in growing seasons in 2021, 2022, and 2023 in the scab field. For each growing season, the 384 clones in the panel were planted each as 5–6 plants per plot. The plot length was 1.5 m, the spacing between plants in each plot was 25 cm, and the spacing between plots was 1 m. The plots were arranged in a randomized design with 1 replication each year. Fertility (100 kg of 15-15-15-2 NPK and Mg) and pesticide (Admire, Capture/Cimegra, Bravo/Orondis/Reason, Superior oil) treatments were applied by following the standard procedures for conventional commercial potato production. No irrigation was performed in this experiment. For each year, planting was performed between 25 May and 15 June, and harvesting was performed in late September. The years 2021 and 2023 were considered normal growing seasons with normal rainfall throughout the growing season, whereas 2022 was considered a mild dry season, with two short drought episodes of 8 days each (20 July to 28 July and 31 July to 7 August) at tuber initiation and bulking stages, and a long episode of 19 days later in the season (28 August to 15 September) (Supplementary Figure S5). Potatoes were harvested using a 3-point lift mounted vegetable harvester (Spudnik Equipment Co., Blackfoot, ID, USA) for the small plot, bagged, and stored at 5 °C, 50% humidity until processing in late October.

To assess common scab symptoms on the tubers, tubers from each sample were washed to remove any soil debris, and the scab reactions were graded according to the Canadian Food Inspection Agency’s fresh fruit or vegetable grade requirement. More specifically, rating was performed as the incidence expressed as a % of the affected tubers, a % of surface coverage in the affected tubers, and severity was rated on a scale of 1 to 5.

### 4.3. Descriptive Statistical Analyses

GWAS computing as conducted in winter/spring 2023 using 2021 and 2022 phenotypic data, and a third-year field trial was initiated and conducted in the summer of 2023. Hence, descriptive statistical analyses were performed on two sets of scab-reaction data, one collected from the 384 clones over two years (2021 and 2022), and one collected over the three years (2021–2023). Descriptive statistical analyses were performed in GeneStat (version 24.1 for Windows) to test the differences between factors, clones, and years, using a mixed model, with clone as the fixed effect and year as the random effect, focusing on clone differences as the main effect. Means from the mixed-model analysis were used to perform hierarchical clustering using Euclidean distances, with clones clipped at 95% to generate most differentiated clusters used as the basis for PCA analyses. The significance for fixed terms was further evaluated using a Wald statistics test (*p* < 0.05). Thereafter, the data were log-transformed for normalization as required in R, version 4.2.0, and the means and BLUPs (best linear unbiased predictions) of the lines for each scab trait were calculated for the two years using R library “Phenotype” version 0.1.0 (https://rdrr.io/cran/Phenotype/man/blup.html (accessed on 18 February 2023) [98,99]. Whenever possible, phenotype diagnostics through distribution plots were generated using GAPIT version 2 [100]. Principal component analysis was performed for population structure and kinship, and the covariates were used for QTN/QTL mapping using the Genome Association and Prediction Integrated Tool Mixed Linear Model (GAPIT-MLM) GWAS, version 3 [70,74,100,101], and the restricted two-stage multi-locus multi-allele genome-wide association study (RTM-GWAS) statistical models [82]. Mean and BLUP values were used for GWAS analyses.

### 4.4. Genotyping

#### 4.4.1. DNA Extraction and Genotyping by Sequencing

DNA extraction and genotyping were performed as described by Fofana et al. [51], and the raw data have been released under SRA accession PRJNA1032882 in the NCBI database [51].

#### 4.4.2. SNP Variant Data Analyses and Population Genetic Structure

Analyses of the raw FASTQ read files, read alignment, and mapping against the potato reference genome (http://spuddb.uga.edu/dm_v6_1_download.shtml, accessed on 18 February 2023) [102] were as described by Fofana et al. [51]. In short, for SNP call, a GQ > 20 and read depth > 5 were set as thresholds to retain a genotype call. SNPs with a call rate < 50%, SNPs for which > 95% of the genotype calls were identical, and SNPs where a minor allele frequency (MAF) is <5% were filtered out. As reported by Fofana et al. [51], the ‘cleaned high-quality dataset’ was used to estimate the decay of linkage disequilibrium (LD) within a sliding window size distance of 10,000 kb for the average LD plot, and the whole chromosome as a sliding window size for LD by chromosome (https://www.cog-genomics.org/plink/1.9/ld, accessed on 18 February 2023), and the SNP coordinates were converted to the chromosome scale of the potato reference genome using GATK version 4.4.0.0 [103]. Using Plink 1.9 [104]; (https://www.cog-genomics.org/plink/1.9/ld, accessed on 18 February 2023), further pruning was performed to produce uncorrelated ‘pruned markers’, resulting in no pair of markers on the same chromosome having a square correlation coefficient *r*^2^ > 0.2. Using GAPIT in *R* [70,74,100,101,105,106], the genetic structure among the 384 accessions was computed using cleaned SNPs distributed across the 12 potato chromosomes as previously described by Fofana et al. [51].

#### 4.4.3. Genome-Wide Association Mapping of Common Scab Traits

As described by Fofana et al. [51], genome-wide trait-to-genotype association mapping was conducted using the GAPIT-mixed linear model (GAPIT-MLM) of GAPIT package, version 3 [70,74,100,101], and the haplotype block-based RTM-GWAS [82] statistical models. The “cleaned” SNP dataset and the three independent trait datasets (trait means, trait BLUPs, and traits separated by years) were used as inputs for association mapping using the GAPIT mixed linear model (MLM) [101] algorithm, with 3 principal components, as indicated by a Scree plot drawn in GAPIT R package. Significant trait-to-marker association thresholds were set as false discovery rates (FDRs)/Benjamani–Hochberg (B and H)-adjusted *p*-value < 0.05. Manhattan plots were produced to summarize and display the GWAS outcomes. Using GAPIT, quantile–quantile (Q˗Q) plots were produced to test the ability of the GWAS statistical models to assess accuracy and to minimize false-positive associations, as described and as reported by Garcia et al. (2019) [107]. For each individual, the number of alleles at QTL that increase the trait value was counted, and the correlation between the number of trait-increasing alleles and trait value was determined using GAPIT MLM. After excluding the missing genotype calls from the calculation, the correlation between the proportion of alleles at QTL (pQTN) that are trait-increasing and the trait value was also determined.

Further, RTM GWAS QTL/QTN mapping was also used on the three independent trait datasets (trait means, trait BLUPs, and traits separate) as in GAPIT. The significant SNP loci were reported at FDR < 0.05, and Manhattan plots and significant QTN/QTL were generated at an FDR adjusted *p*-value < 0.05 as described by Fofana et al. [51]. Similarly to the pQTN analysis using GAPIT MLM, pQTN analysis was performed using the RTM-GWAS model as previously described [51].

Significant (*p* < 0.05) QTN from the GAPIT MLM-GWAS analyses were further evaluated, and those found in at least two of the studied scab traits were retained and compared with the QTNs/QTLs derived from the RTM-GWAS model. Mann–Whitney non-parametric *U*-tests (*p* < 0.05) were performed to remove the potential false-positive QTNs using Blue Sky statistics (https://www.blueskystatistics.com/, accessed on 18 February 2023).

To test the QTN/QTL effect on trait means, the number of QTNs/QTLs with positive-effect alleles (PQTLs) in all accessions was registered. A PQTL is defined as one that significantly increases the phenotypic value of a scab trait based on the Mann–Whitney non-parametric *U* test (*p* < 0.05). We compared whether the phenotypic differences for the traits assessed are attributable to the accumulation of PQTL between two contrasting subsets of accessions (>95th) represented by the 20 with the highest and the 20 with lowest number of PQTLs. Statistical differences between the subset of accessions were tested with a Student’s *t*-test (*p* < 0.05). Heatmap analysis was carried out for the contrasting accessions and PQTL using TBTools software, version V2.119 [108]. The PQTLs were codified as “1”, while the alternate allele was coded as “0”. The options “Euclidean distances” and “Average” were used as the metric parameter and the linkage method, respectively. Violin plots were generated using Blue Sky statistics (https://www.blueskystatistics.com/, accessed on 14 September 2024).

### 4.5. Candidate Genes Identification

Candidate genes co-located within a window of 20,000 bp on either side of the significant QTNs were scanned as previously described [75] and annotated based on the most recent release of the potato reference genome [102].

## Figures and Tables

**Table 1:** Range distributions of common scab rating in two phenotypic datasets. Lower (0–40%), medium (41–70%), and high (70–100) incidence; lower (0–1.5), medium (1.6–2.5), and high (2.6–5) severity; lower (1–10%), medium (11–30%), and high (31–100) surface coverage.

Datasets	Scab Traits	Mean Rating			% Clones per Category		
		Lower	Medium	High	Low	Medium	High
2021–2022	Incidence	0 ± 0	52.60 ± 9.6	98.75 ± 2.45	0	1	99
	Severity	1.33 ± 0.24	2.21 ± 0.26	3.62 ± 0.62	24.0	43	33
	Coverage	6.93 ± 2.24	18.22 ± 5.37	41.18 ± 8.31	35.1	55	9.82
2021–2023	Incidence	26.07 ± 12.49	60.41 ± 8.5	92.42 ± 8.43	3.1	11.62	85.27
	Severity	1.20 ± 0.28	2.13 ± 0.27	3.33 ± 0.48	44.7	34.3	20.93
	Coverage	6.17 ± 2.60	15.47 ± 4.49	41.09 ± 14.36	52.97	42.89	4.14

**Table 2:** Allelic effect of 52 significant QTN/QTLs based on the Mann–Whitney non-parametric *U* test (*p* < 0.05) for scab coverage, severity, and incidence.

Traits	Blup Scab Coverage QTLs	Leading QTN *	Genotype Mean		Effect	*p* Value	Pleiotropic
Coverage	Schr01_1728598	Schr01_1728598	AA = 2.75	**GG** = 2.29	−0.46	0.002	Coverage/incidence
	Block_chr01_3114493_3114575	Schr01_3114566	**GG** = 2.35	TT = 2.96	−0.61	0.0003	Coverage/incidence
	Schr03_52163558	Schr03_52163558	**AA** = 2.29	GG = 2.61	−0.32	0.019	
	Schr03_53916615	Schr03_53916615	**AA** = 2.51	TT = 2.69	−0.18	0.038	Coverage/severity/ incidence
	Block_chr03_58019530_58059755	Schr03_58059755	**GG** = 2.63	TT = 3.09	−0.46	0.031	
	Schr04_4988962	Schr04_4988962	GG = 2.62	**TT** = 2.13	−0.49	0.019	
	Block_chr04_63320301_63320382	Schr04_63320547	**AA** = 2.49	GG = 2.88	−0.39	0.021	Coverage/severity
	Schr04_65832234	Schr04_65832234	**CC** = 2.50	GG = 2.77	−0.27	0.036	Coverage/incidence
	Schr04_65832323	Schr04_65832323	AA = 2.78	**GG** = 2.52	−0.26	0.041	Coverage/severity/ incidence
	Block_chr04_68993437_68993521	Schr04_68993521	**AA** = 2.47	TT = 2.69	−0.22	0.015	Coverage/severity/ incidence
	Block_chr05_165634_253736	Schr05_165689	CC = 2.78	**TT** = 2.39	−0.39	0.002	
	Block_chr05_800551_800594	Schr05_800551	**CC** = 2.30	TT = 2.65	−0.35	0.011	Coverage/severity/ incidence
	Schr09_6988187	Schr09_6988187	AA = 3.07	**GG** = 2.36	−0.71	0.044	Coverage/severity/ incidence
	Schr09_52765405	Schr09_52765405	AA = 2.76	GG = 2.26	−0.50	0.0001	
	Block_chr09_59556748_59556862	Schr09_59556862	**AA** = 2.37	CC = 2.65	−0.28	0.019	Coverage/incidence
	Block_chr09_66093163_66093283	Schr09_66093163	**AA** = 2.27	TT = 2.59	−0.32	0.019	Coverage/incidence
	Block_chr10_50975501_50975603	Schr10_50975603	**AA** = 2.36	CC = 2.80	−0.44	0.002	Coverage/incidence
	Block_chr11_39049243_39049264	Schr11_39049264	AA 2.66	**GG** = 2.42	−0.24	0.014	Coverage/severity/ incidence
	Block_chr11_39049290_39049364	Schr11_39049364	AA = 2.68	**GG** = 2.42	−0.26	0.007	Coverage/severity
	Schr12_1537392	Schr12_1537392	**AA** = 2.28	GG = 2.57	−0.29	0.047	
	Blup Scab Severity QTLs						
Severity	Schr03_53916615	Schr03_53916615	**AA** = 0.44	TT = 0.73	−0.29	0.001	Coverage/severity/ incidence
	Schr03_53916666	Schr03_53916666	**AA** = 0.53	TT = 0.77	−0.24	0.025	
	Block_chr03_54034507_54034531	Schr03_54034531	**AA** = 0.04	GG = 0.58	−0.54	0.014	
	Block_chr03_54180732_54210225	Schr03_54180802	**AA** = 0.13	GG = 0.56	−0.43	0.027	Severity/incidence
	Block_chr04_63320301_63320382	Schr04_63320301	AA = 0.72	**GG** = 0.08	−0.64	0.0001	Severity/coverage
	Schr04_65832323	Schr04_65832323	AA = 0.76	**GG** = 0.59	−0.17	0.043	Coverage/severity/ incidence
	Block_chr04_68993437_68993521	Schr04_68993521	**AA** = 0.49	TT = 0.71	−0.22	0.012	Coverage/severity/ incidence
	Block_chr05_800551_800594	Schr05_800551	**CC** = 0.44	TT = 0.59	−0.15	0.037	Coverage/severity/ incidence
	Schr06_1158879	Schr06_1158879	**CC** = 0.16	TT = 0.69	−0.53	0.0009	
	Schr07_381002	Schr07_381002	GG = 0.78	**TT** = 0.51	−0.27	0.014	
	Block_chr08_58892718_58892767	Schr08_58892711	CC = 0.74	**TT** = 0.18	−0.56	0.0001	Severity/incidence
	Schr09_6988187	Schr09_6988187	AA = 0.85	**GG** = 0.45	−0.40	0.042	Coverage/severity/ incidence
	Block_chr11_39049243_39049264	Schr11_39049243	AA = 0.76	**GG** = 0.40	−0.36	2.7 × 10^−5^	Coverage/severity/ incidence
	Block_chr11_39049290_39049364	Schr11_39049364	AA = 0.77	**GG** = 0.39	−0.38	1.4 × 10^−5^	Severity/coverage
	Block_chr11_41465706_41465874	Schr11_41465706	**AA** = 0.16	GG = 0.59	−0.43	0.044	
	Block_chr12_1934225_1934235	Schr12_1934235	AA = 0.90	**GG** = 0.51	−0.39	0.0002	
	Blup Sca Incidence QTLs						
Incidence	Schr01_1728598	Schr01_1728598	AA = 96.9	**GG** = 89.8	−7.1	7.4 × 10^−5^	Incidence/coverage
	Block_chr01_2150739_2150762	Schr01_2150762	**CC** = 92.2	TT = 94.3	−2.1	0.01	
	Block_chr01_3114493_3114575	Schr01_3114566	**GG** = 90.9	TT = 97.5	−6.6	0.002	Incidence/coverage
	Schr03_53916615	Schr03_53916615	**AA** = 91.6	TT = 94.3	−2.7	0.015	Coverage/severity/ incidence
	Block_chr03_54180732_54210225	Schr03_54180802	**AA** = 88.1	GG = 93.9	−5.8	0.014	Incidence/severity
	Schr04_65832234	Schr04_65832234	**CC** = 92.4	GG = 95.5	−3.1	0.035	Incidence/coverage
	Schr04_65832323	Schr04_65832323	AA = 95.8	**GG** = 92.5	−3.3	0.031	Coverage/severity/ incidence
	Block_chr04_68993437_68993521	Schr04_68993521	**AA** = 91.9	TT = 94.4	−2.5	0.046	Coverage/severity/ incidence
	Block_chr05_800551_800594	Schr05_800655	AA = 94.0	**TT** = 89.2	−4.8	0.009	Coverage/severity/ incidence
	Block_chr08_58892718_58892767	Schr08_58892711	CC = 93.6	**TT** = 91.7	−1.9	0.043	Incidence/severity
	Schr09_6988187	Schr09_6988187	AA = 96.1	**GG** = 92.3	−3.8	0.033	Coverage/severity/ incidence
	Block_chr09_59556748_59556862	Schr09_59556862	**AA** = 92.2	CC = 95.2	−3.0	0.041	Incidence/coverage
	Block_chr09_66093163_66093283	Schr09_66093163	CC = 94.7	**TT** = 89.3	−5.4	0.015	Incidence/coverage
	Block_chr10_50975501_50975603	Schr10_50975603	**AA** = 88.6	CC = 96.3	−7.7	0.002	Incidence/coverage
	Block_chr11_39049243_39049264	Schr11_39049264	AA = 94.1	**GG** = 91.6	−2.5	0.041	Coverage/severity/ incidence
	Block_chr12_1931979_1932004	Schr12_1932052	**CC** = 85.9	TT = 93.2	−7.3	0.032	

* Quantitative trait nucleotide showing the highest *p* value within an LD block estimated either with GAPIT version 3 2022-11-08 or RTM-GWAS software, version 2022.0-rc2. Bold letters represent genotypes with positive effects for the trait.

**Table 3:** Cross-reference comparison of selected genes playing roles in plant defense and detected through GWAS and RNAseq transcriptional studies of potato/common scab pathosystem.

Description and Function of Selected Genes Detected by GWAS and RNAseq		Current GWAS Study	RNAseq Detected by Fofana et al. [34]
Gene Description	Gene Functions	GWAS Gene ID	RNAseq Protein/KEEG ID
Receptor-like protein Cf-9	Pattern-triggered immunity (PTI) signaling induced	Soltu.DM.01G004470.1	XP_015161059.1 XP_006358586.1, XP_006358588.1, XP_006365630.1 XP_006356443.1
Transcription factor bHLH148	bHLH transcription factor Negatively regulates brassinosteroid signaling	Soltu.DM.03G027480.1	XP_006357746.1 (bHLH18) XP_006357756.2 (BHLH93)
Transcription factor bHLH62 (basic helix–loop–helix protein 62) (AtbHLH62) (bHLH 62) (transcription factor EN 85) (bHLH transcription factor bHLH062)	Functions redundantly with IBH1/BHLH158 in a regulation node; acts as transcriptional repressor that negatively regulates cell and organ elongation in response to gibberellin (GA) and brassinosteroid (BR) signaling	Soltu.DM.12G002380.1	XP_006357746.1 (bHLH18) XP_006357756.2 (BHLH93) Stress responses
Transcription factor IBH1-like 1 (AtIBL1) (BHLH transcription factor eta) (bHLH eta)	Acts as transcriptional repressor that negatively regulates cell and organ elongation in response to gibberellin (GA) and brassinosteroid (BR) signaling	Soltu.DM.12G002380.1	
Serine/threonine-protein kinase CDG1 (EC 2.7.11.1) (protein CONSTITUTIVE DIFFERENTIAL GROWTH 1)	Mediates BR signal transduction from BRI1 receptor kinase to BSU1 phosphatase. Involved in the positive regulation of brassinosteroid (BR) signaling and plant growth.	Soltu.DM.04G025270.1	XP_015167936.1 XP_006362170.1 (CBL-interacting SER/threonine-protein kinase XP_006343494.1, XP_006343495.1 (Aurora-3) XP_015166356.1 XP_006356742.1 XP_006358074.2, XP_015169269.1, XP_015169270.1 XP_006367657.1 (CDL1)
Transcription factor MYB73	Transcription factor that functions in stress response. Acts in phenylpropanoid/flavonoid pathways	Soltu.DM.01G002070.1	XP_006359691.1 XP_006345132.1 XP_006352842.1 XP_006366298.1 (revesratrol methyltranferase)
Putative late blight resistance protein homolog R1B-12	Confers resistance to late blight (Phytophthora infestans) carrying the avirulence gene Avr1; resistance proteins guard the plant against pathogens that contain an appropriate avirulence protein via an indirect interaction with this avirulence protein; this triggers a defense system including the hypersensitive response, which restricts pathogen growth	Soltu.DM.06G000830.1 Soltu.DM.12G002390.1 Soltu.DM.06G001240.1 Soltu.DM.12G002410.1	XP_015158514.1, XP_015158515.1 XP_006356446.1 XP_015167139.1, XP_015167140.1 XP_015167121.1 XP_015162516.1 XP_015161246.1
Subtilisin-like protease 4 (SbtM4) (Subtilase 4) (EC 3.4.21.-)	Required for arbuscular mycorrhiza (AM) development during AM symbiosis with AM fungi (e.g., Glomeromycota intraradices)	Soltu.DM.09G018920.1 Soltu.DM.08G026440.1	XP_006365833.1
Xyloglucan galactosyltransferase MUR3	Catalyzes xyloglucan endohydrolysis (XEH) and/or endotransglycosylation (XET).	Soltu.DM.09G018910.1	XP_006365833.1 XP_006354588.1 NP_001274880.1 XP_006349017.1
Lignin-forming anionic peroxidase (EC 1.11.1.7) (TOPA)	Lignin-forming anionic peroxidase	Soltu.DM.01G001840.1 Soltu.DM.01G002950.1	XP_006366125.1 (ascorbate peroxidase)
Heparanase-like protein 2 (EC 3.2.-.-)	Endoglycosidase with cell surface- and extracellular matrix-degrading enzyme; cleaves heparan sulfate proteoglycans	Soltu.DM.03G029620.1 Soltu.DM.06G025660.1	
Cytochrome P450 734A6 (EC 1.14.-.-)	Involved in brassinosteroid (BR) inactivation and regulation of BRs homeostasis	Soltu.DM.03G034600.1	XP_006367342.1 (CYP 83B1) XP_006359799.1 1 (CYP 71A1) XP_006339107.1 (CYP 76A2) XP_015167656.1 (CYP 82C4) XP_006359946.1 XP_006359946.1 (CYP71D11) XP_015164889.1 (CYP72A219) XP_006364811.1 (CYP82D47) XP_006339516.2 (CYP704C1) XP_006351638.1 (CYP94A2)
Serine/threonine-protein kinase D6PKL2 (EC 2.7.11.1) (D6 protein kinase-like 2) (Serine/threonine-protein kinase AtPK5)	Regulates the auxin transport activity of PIN auxin efflux facilitators	Soltu.DM.11G021470.1	XP_006341527.1 (auxin) XP_006343229.1 (auxin-repressed protein) XP_006353769.1 auxin-responsive protein (SAUR72) XP_006342573.1 (auxin-inducible protein)
Calmodulin-4 (CaM-4)	Calmodulin mediates the control of a large number of enzymes, ion channels, and other proteins by Ca(2+); among the enzymes to be stimulated by the calmodulin-Ca(2+) complex are a number of protein kinases and phosphatases; activates MPK8 through direct binding and in a calcium-dependent manner.	Soltu.DM.03G029780.1	XP_006367976.1
Transcription factor MYB73 (Myb-related protein 73) (AtMYB73)	Transcription factor that functions in stress response; acts in response to auxin, activates the transcription of the auxin-responsive gene IAA19; IAA19 transcription activation by MYB73 is enhanced by direct interaction between MYB73 and PYL8	Soltu.DM.01G002070.1 Soltu.DM.11G021480.1	NP_001275604.1 (WRKY TF) XP_015159146.1, XP_015159147.1 (bZIP TF) XP_006345791.1, XP_006345792.1 (GATA)
Rhamnogalacturonan I rhamnosyltransferase 1 (EC 2.4.1.351) (O-fucosyltransferase 3) (O-FucT-3) (O-fucosyltransferase family protein)	Glycosyltransferase activity, involved in the formation of rhamnogalacturonan I (RG-I) oligosaccharides in the seed coat mucilage	Soltu.DM.05G000090.1	XP_006365231.2 XP_006346094.1 XP_015163068.1 (Arabinogalactan)
UDP-arabinopyranose mutase 3 (OsUAM3) (EC 5.4.99.30) (reversibly glycosylated polypeptide 3) (UDP-L-arabinose mutase 3)	UDP-L-arabinose mutase activity, involved in the biosynthesis of cell wall non-cellulosic polysaccharides	Soltu.DM.05G001020.1	XP_015163068.1 (Arabinogalactan)
Ubiquitin-like domain-containing protein CIP73 (CCaMK-interacting protein of approximately 73 kDa)	Involved in root nodulation; required for root nodule organogenesis after infection by symbiotic rhizobia	Soltu.DM.06G008090.1	XP_015162063.1 XP_006367213.1 XP_015163563.1 XP_006359581.1, XP_015169967.1
Pectinesterase (PE) (EC 3.1.1.11) (Pectin methylesterase)	Acts in the modification of cell walls via demethylesterification of cell wall pectin [52]	Soltu.DM.09G023660.1	
Endoglucanase 17 (EC 3.2.1.4) (Endo-1,4-beta glucanase 17)	Cellulose degration/metabolism	Soltu.DM.09G023670.1	XP_006356407.1 XP_006339416.1
LRR receptor-like serine/threonine-protein kinase GSO1 (EC 2.7.11.1) (protein GASSHO 1) (protein SCHENGEN 3)	In coordination with GSO2, regulates root growth through control of cell division and cell fate specification; involved in the regulation of suberin accumulation in the endodermis [53]	Soltu.DM.12G026650.1	XP_015159898.1 (FEI), XP_006356742.1, XP_006358074.2, XP_006348920.1, XP_015169250.1, XP_006354051.1
Protein ETHYLENE INSENSITIVE 3	Transcription factor acting as a positive regulator in the ethylene response pathway	Soltu.DM.12G026660.1	NP_001275149.1 (ACC oxidase) XP_006347933.1 (ethylene-responsive TF ERF034) XP_006339057.1 (ethylene-responsive TF RAP2) XP_015162960.1 (ethylene-insensitive) XP_006365957.1 (NEP1-interacting protein)
Disease resistance protein ADR1 (activated disease resistance protein 1)	Disease resistance (R) protein that mediates resistance against Hyaloperonospora parasitica in a salicylic acid-dependent manner; mediates resistance against Erysiphe cichoracearum in both salicylic acid-dependence and partially NPR1-dependence; resistance proteins guard the plant against pathogens that contain an appropriate avirulence protein; triggers a defense system, including the hypersensitive response	Soltu.DM.04G034340.1	XP_015164732.1
Xylulose kinase (Xylulokinase) (EC 2.7.1.17)	Phosphorylates D-xylulose to produce D-xylulose 5-phosphate, a molecule that may play an important role in the regulation of glucose metabolism and lipogenesis	Soltu.DM.04G031750.1	XP_006354009.1 (xylosidase)
Sesquiterpene synthase 9 (SlTPS9) (Terpene synthase 9) (Beta-myrcene synthase TPS9) (EC 4.2.3.15) (Germacrene C synthase TPS9) (EC 4.2.3.60) (Limonene synthase TPS9) (EC 4.2.3.-) (Sesquiterpene synthase 1) (Terpinolene synthase TPS9) (EC 4.2.3.113)	Involved in the biosynthesis of germacrene C, one of the most abundant sesquiterpenes in tomato leaf [54]; produces mainly germacrene C, and germacrene A, B, and D; no or low activity with geranylgeranyl diphosphate (GGPP) [55]; acts with low efficiency as a monoterpene synthase with geranyl diphosphate (GPP) as a substrate, thus producing beta-myrcene, limonene, and terpinolene [55,56]	Soltu.DM.09G029980.1	XP_015167610.1 (zeatin xylotranferase) XP_006362006.1, XP_006362007.1, XP_006362008.1 Ssqualene synthase) XP_006368148.1 (geranylgeranyl transferase) NP_001275191.1, XP_006352913.1, XP_015166573.1 XP_006352698.1 (tropinone reductase)
Xyloglucan endotransglucosylase/hydrolase 2 (EC 2.4.1.207) (Brassinosteroid-regulated protein BRU1)	Catalyzes xyloglucan endohydrolysis (XEH) and/or endotransglycosylation (XET); cleaves and religates xyloglucan polymers, essential for the primary cell wall, and cell wall formation	Soltu.DM.09G030000.1	XP_006349017.1 XP_006354588.1
Wall-associated receptor kinase 2 (EC 2.7.11.-)		Soltu.DM.10G019560.1 Soltu.DM.10G019550.1	XP_006366426.1 XP_006341994.1 (protin walls are thin1—auxin transport)
Chitin-inducible gibberellin-responsive protein 2	Regulatory role in the early step of oligosaccharide elicitor response, downstream of the membrane-associated high-affinity chitin-binding protein	Soltu.DM.11G000400.1	XP_015161134.1 (chitin binding lectin1) role in immune response against pathogens XP_006364607.2 (chitin binding lectin1)
MACPF domain-containing protein CAD1 (protein CONSTITUTIVELY ACTIVATED CELL DEATH 1) (protein CAD1)	Negatively controls the salicylic acid (SA)-mediated pathway of programmed cell death in plant immunity [57]	Soltu.DM.10G021860.1	XP_015160896.1, XP_006346475.1 Salicylic acid binding protein
Endoribonuclease Dicer homolog 4 (EC 3.1.26.-) (Dicer-like protein 4) (OsDCL4) (protein SHOOT ORGANIZATION 1)	RNA silencing pathway; cleaves double-stranded RNA to produce small interfering RNAs (siRNAs) for selective destruction of complementary RNAs	Soltu.DM.07G000050.1	XP_015166743.1, XP_015166744.1
Cellulose synthase	Cellulose synthesis	Soltu.DM.09G018910.1	XP_006358416.1, XP_015169407.1 XP_015167354.1 XP_015167353.1 XP_006343827.1

**Table 4:** Frequency distribution of diploid potato clones used in this study.

Types	Cross #	Number of Clones	Proportion of Clones (%) *
47 CTL	1	6	12.8
	2	8	17.0
	3	5	10.6
	4	6	12.8
	5	8	17.0
	6	9	19.1
	7	5	10.6
337 EMS	1	42	12.5
	2	65	19.3
	3	40	11.9
	4	34	10.0
	5	48	14.2
	6	43	12.8
	7	65	19.3

* Proportion determined within each type.

## Data Availability

The raw sequencing data had been previously released as PRJNA1032882 in the NCBI database by Fofana et al. [51]. All other relevant data generated or analyzed during this study are provided in full within the published article and its Supplementary Materials.

## Supplementary Materials

The following supporting information can be downloaded at: https://www.mdpi.com/article/10.3390/ijms26031126/s1.
