# Supplementary material for: Genome-Wide Genetic Architecture for Common Scab (Streptomyces scabei L.) Resistance in Diploid Potatoes
Source: Int J Mol Sci. 2025 Jan 28;26(3):1126. doi: 10.3390/ijms26031126 (PMC11818057; doi:10.3390/ijms26031126)
Supplement: Supplementary file 1 [file ijms-26-01126-s001.zip › Supplementary Figures S1 - S5.pdf]

Supplementary Figures S1 - S5

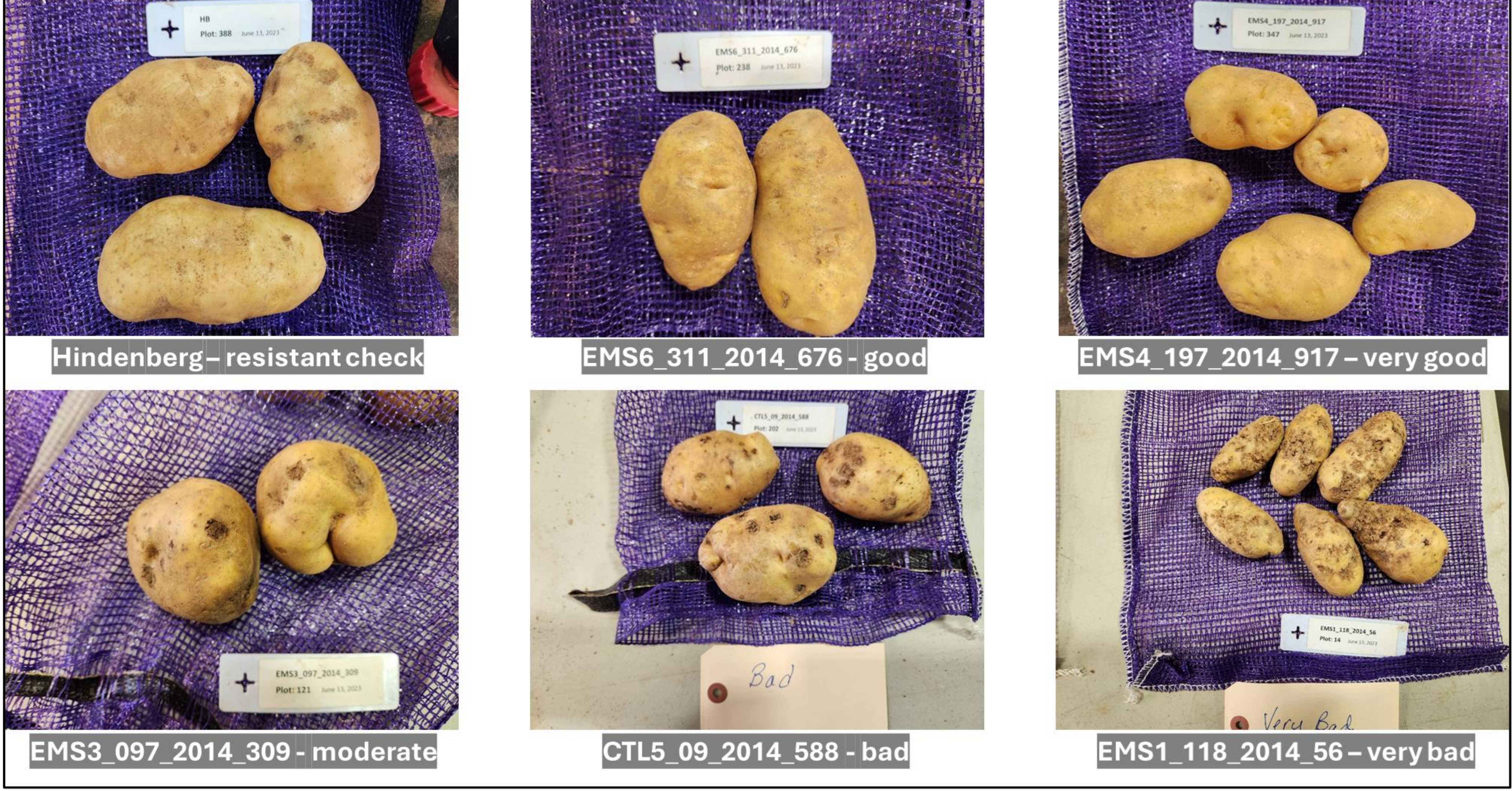

Suppl Figure S1. Comparative phenotypic reactions observed with the Check (Hindenburg) and five diploid potato clones.

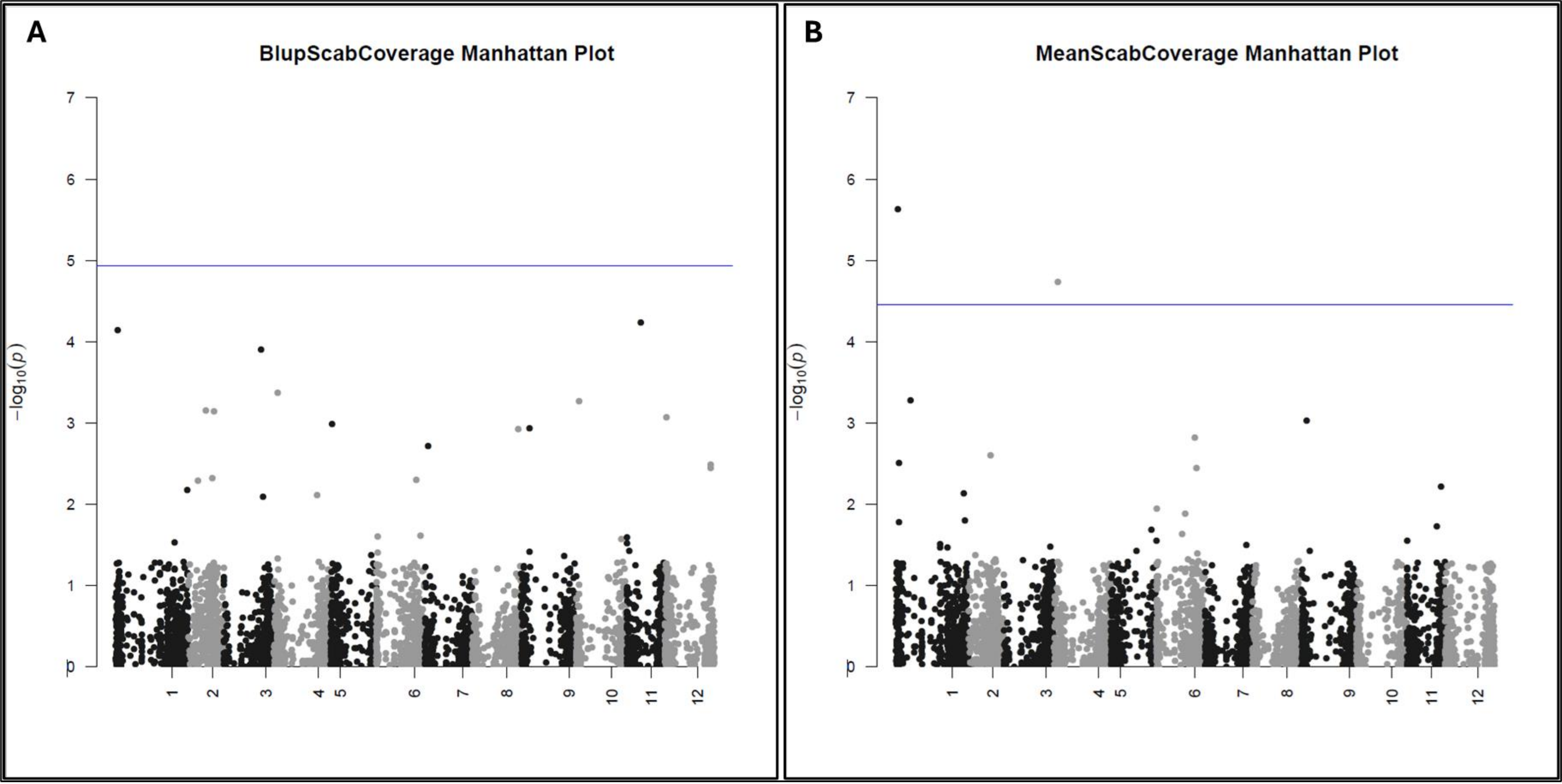

Suppl Figure S2A,B. Manhattan plot showing QTN/QTL and chromosomal regions associated with common scab coverage trait using the RTM-GWAS model. A) BLUP-based Manhattan plot; B) Mean-based Manhattan plot. The blue line indicates the second FDR threshold cut off <0.05.

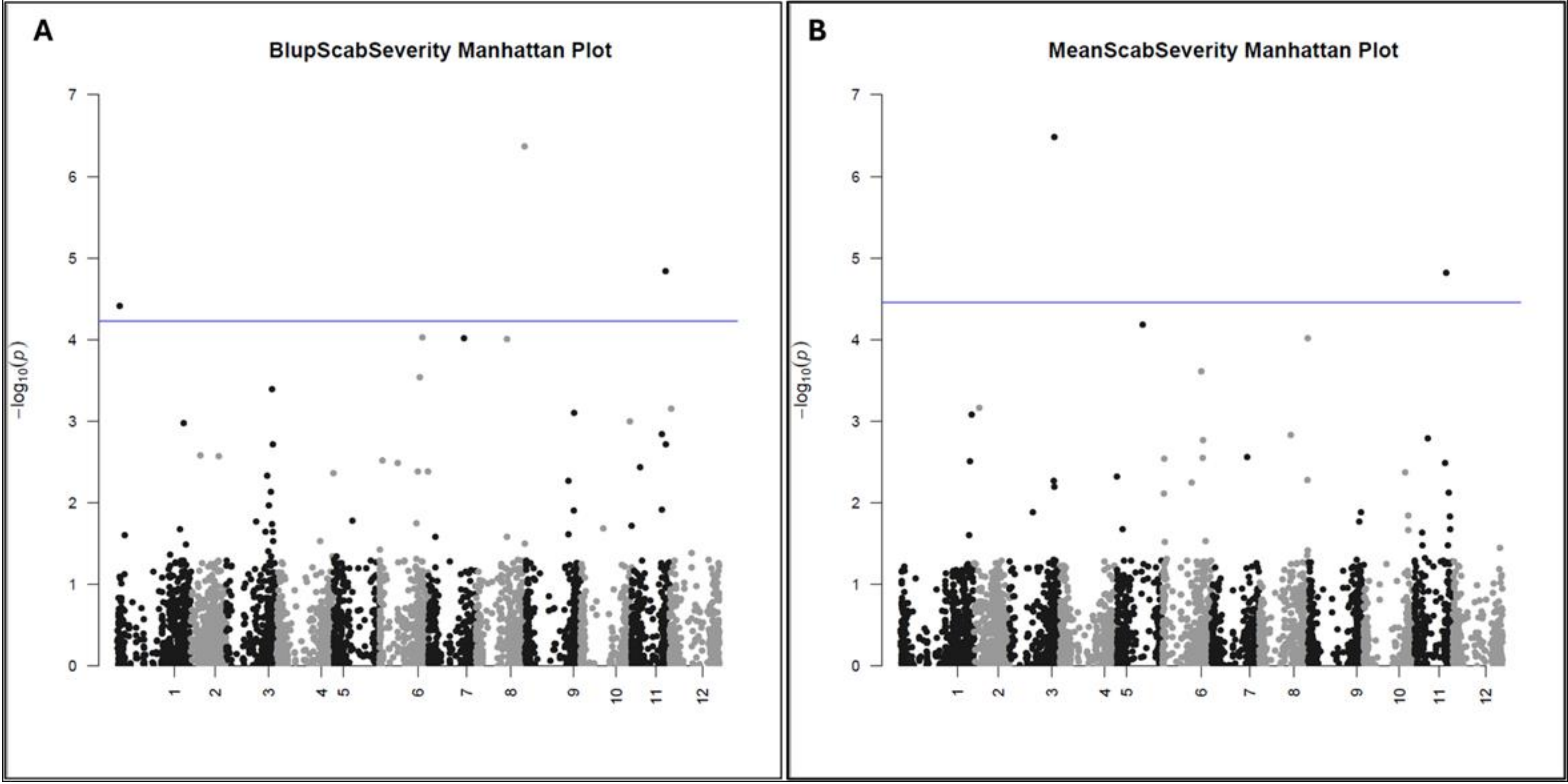

Suppl Figure S3A,B. Manhattan plot showing QTN/QTL and chromosomal regions associated with common scab severity trait using the RTM-GWAS model. A) BLUP-based Manhattan plot; B) Mean-based Manhattan plot. The blue line indicates the second FDR threshold cut off <0.05.

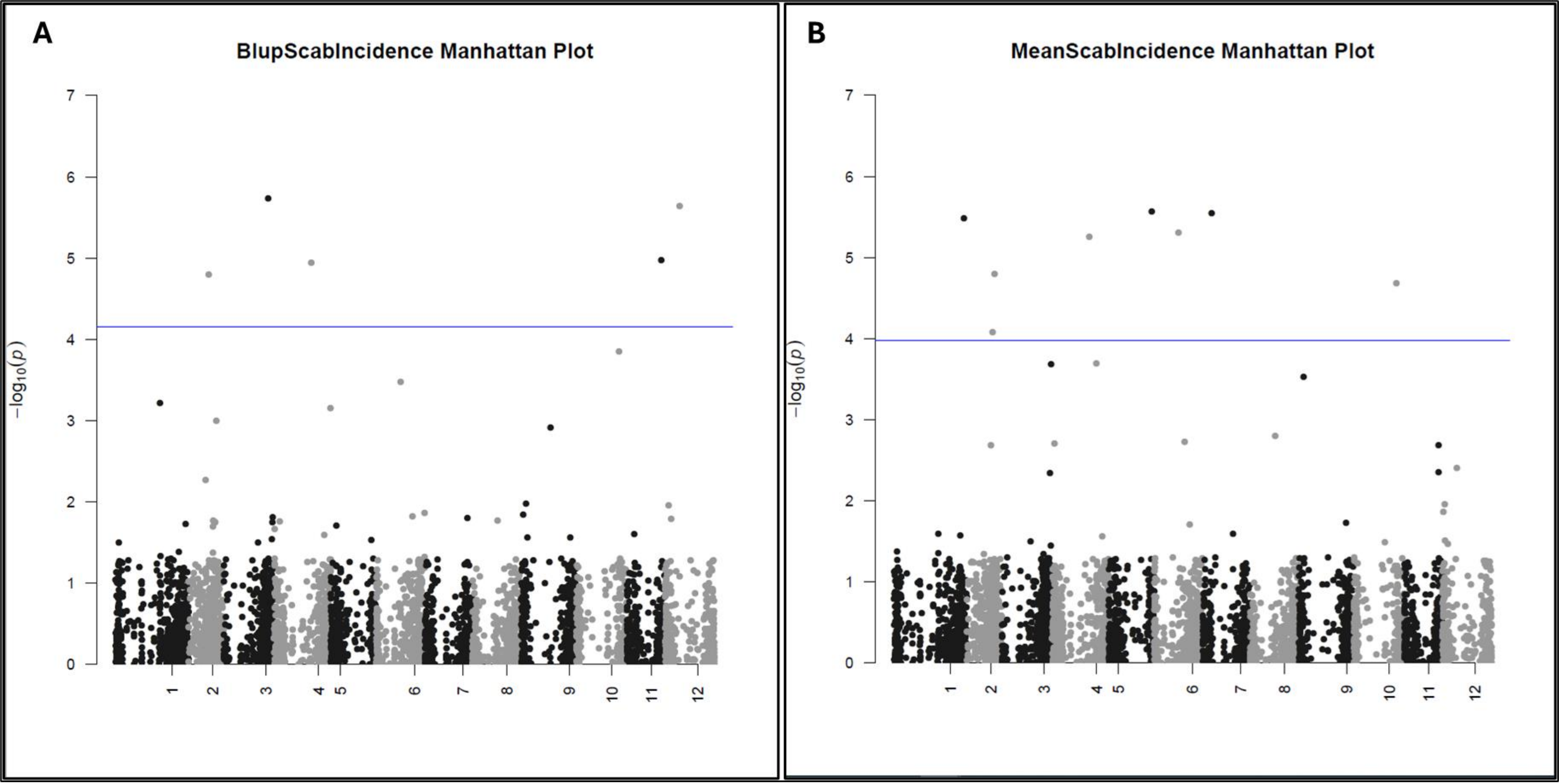

Suppl Figure S4A,B. Manhattan plot showing QTN/QTL and chromosomal regions associated with common scab incidence trait using the RTM-GWAS model. A) BLUP-based Manhattan plot; B) Mean-based Manhattan plot. The blue line indicates the second FDR threshold cut off <0.05.

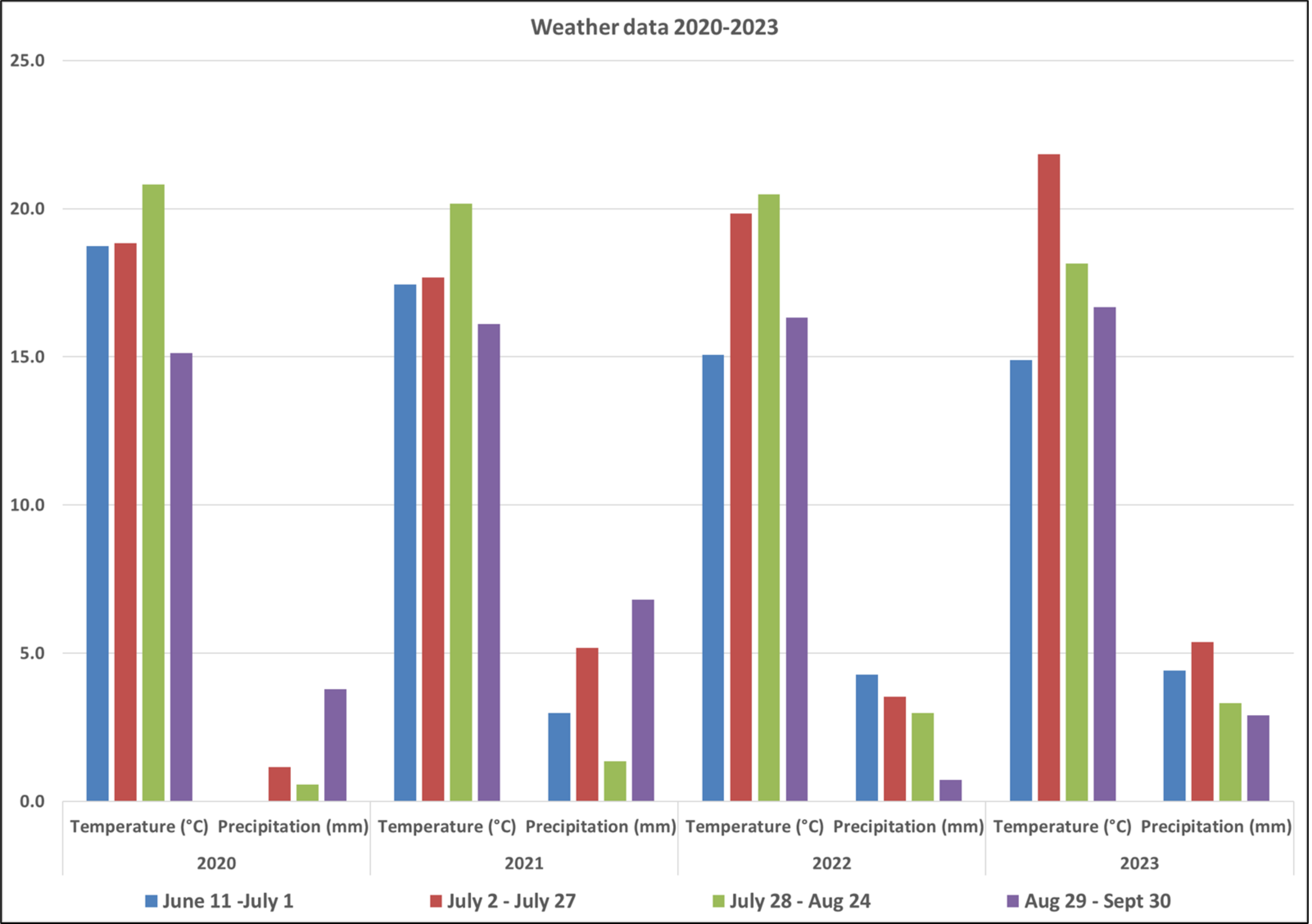

Suppl Figure S5. Temperature (°C) and precipitation (mm) data during growing seasons in years 2020 to 2023.
